# Is hyperlactatemia a useful screening marker of severe sepsis in hemato-oncologic malignant patients?

**DOI:** 10.1186/2197-425X-3-S1-A300

**Published:** 2015-10-01

**Authors:** S Park, J Huh

**Affiliations:** Pulmonary and Critical Care Medicine, University of Ulsan College of Medicine/Asan Medical Center, Seoul, Korea, Republic of

## Introduction

Hyperlactatemia is often associated with poor clinical outcomes. Mortality is increased when lactic acidosis with low-flow states or sepsis. For improving poor prognosis, early detection and intervention of hyperlactatemia are important. There are call for monitoring blood lactate level. However, hyperlactatemia is often associated with type II lactic acidosis in hemato-oncologic malignant patients.

## Objectives

We investigate there are difference in blood lactate level between severe sepsis and Type II lactic acidosis in hemato-oncologic malignant patients.

## Methods

Medical records of 99 patients in hemato-oncologic malignant patients admitted at Asan Medical Center from January 1, 2013 to June 31, 2013 was reviewed. Above 4 mmol/L blood lactate were screening.

## Results

Among 99 patients with hyperlactatemia, 35 patients showed severe sepsis, 64 patients showed Type II lactic acidosis. There was no significant difference in basal characteristics. Blood lactate level was no significant difference (6.76 mmol/L in severe sepsis and 6.10 mmol/L in type II lactic acidosis, P-value = 0.301). There are significant difference in blood lactate level between oncologic malignant patients and hematologic malignant patients (6.63 mmol/L and 5.48 mmol/L, P-value = 0.029). In oncologic malignant patients, blood lactate level was no significant difference between severe sepsis and type II lactic acidosis (7.26 mmol/L and 6.32 mmol/L, P-value = 0.291). In hematologic malignant patients, blood lactate level was no significant difference between severe sepsis and type II lactic acidosis (5.67 mmol/L and 5.32 mmol/L, P-value = 0.639)

## Conclusions

Hyperlactatemis is associated with poor prognosis. It is important blood lactate level screening in severe sepsis. However, blood lactate level is not useful with hemato-oncologic malignant patients for sepsis screening. Other indicators to suggest sepsis are more useful sepsis screening tool. For example, other indicator were blood pressure, heart rate, respiratory rate, body temperature, mental status, oxygen requirement and urine output etc.

